# Turning back time: a comprehensive list of interventions that decrease next-generation epigenetic aging clocks in humans

**DOI:** 10.3389/fgene.2026.1836446

**Published:** 2026-05-29

**Authors:** Adiv A. Johnson, David A. Sinclair

**Affiliations:** 1 Tally Health, New York, NY, United States; 2 Paul F. Glenn Center for Biology of Aging Research, Department of Genetics, Blavatnik Institute, Harvard Medical School, Boston, MA, United States

**Keywords:** aging biomarker, biohorology, clinical trials, epigenetic age reversal, epigenetic aging clock, next-generation clock

## Abstract

Epigenetic aging clocks estimate age from DNA methylation patterns and have become central tools in longevity research. More recently, next-generation clocks have been developed to better compensate for the known divergence between chronological age and epigenetic age in ways that relate to lifestyle, health, and age-related disease. Although epigenetic clocks represent investigational biomarkers, these newer models are more strongly associated with all-cause mortality risk than first-generation clocks. As such, interventions that modify them are of interest. To test this, we performed a series of systematic searches and identified 41 human studies reporting the effects of interventions on at least one next-generation epigenetic clock. Our data suggest that a diverse range of pharmaceutical, lifestyle, supplementation, non-pharmaceutical clinical, and psychosocial interventions can decrease epigenetic age, including exercise, a plant-rich diet, the GLP-1 receptor agonist semaglutide, caloric restriction, ketamine, omega-3 fatty acids, a multivitamin-multimineral supplement, umbilical cord plasma, and the cholesterol-lowering drug pitavastatin. Nicotinamide riboside, rapamycin, senolytics, and several other interventions showed no detectable effect, whereas plasmapheresis and other therapeutics accelerated epigenetic aging. We also summarize reported effect sizes and compare next-generation clocks with respect to their frequency of use and responsiveness to intervention.

## Introduction

In 2011, Bocklandt and colleagues published a seminal paper demonstrating that chronological age could be accurately predicted using just a handful of DNA methylation sites. Using three methylation sites annotated to the genes *EDARADD*, *ELN*, and *NPTX2*, the authors created a predictive linear model that achieved a Pearson correlation of 0.87 and an average error of 3.5 years in adult saliva (Bocklandt et al., 2011). Two years later, Horvath showed that methylomic data could be used to build a pan-tissue chronological age predictor and described this epigenetic model as an “aging clock” (Horvath, 2013). Earlier that year, Gregory Hannum and his colleagues used DNA methylation information to create a separate blood-based predictor of chronological age (Hannum et al., 2013).

Aging clocks have come a long way since these nascent studies and the science of molecular age prediction has been dubbed “biohorology” (Galkin et al., 2020). Importantly, more capable next-generation clocks have been developed and in use since 2018 (Levine et al., 2018). Unlike the earlier, first-generation models that were purely trained to predict chronological age accurately, next-generation clocks undergo strategic, multi-step training programs to produce an age estimate that is significantly associated with age-related outcomes, lifestyle, and/or health. In addition to being more strongly associated with all-cause mortality risk in longitudinal data, next-generation models are more adept at capturing clinically relevant signals and are more responsive to interventions (Johnson and Shokhirev, 2025).

To date, myriad approaches have been employed to generate next-generation epigenetic aging clocks. DNAm PhenoAge (Levine et al., 2018), for example, used DNA methylation to predict the output of a mortality-associated model based on clinical blood proteins. Models like GrimAge (Lu et al., 2019), GrimAge2 (Lu et al., 2022), and OMICmAge (Chen et al., 2026) involve using DNA methylation to estimate protein, metabolite, and/or clinical parameters such as hemoglobin A1C and C-reactive protein. These methylation-based estimators have been referred to as epigenetic biomarker proxies (Chen et al., 2026). Conversely, the rate-based clocks DunedinPoAm (Belsky et al., 2020) and DunedinPACE (Belsky et al., 2022) are trained on longitudinal biomarker change and output a rate as opposed to an age. Regardless of the approach used, what differentiates a next-generation clock from a first-generation clock is that the training focus is to produce a signal that is relevant to health and, ideally, outcomes.

While epigenetic aging clocks represent exciting research tools, they do have limitations. On their own, they do not sufficiently capture biological age (Johnson and Shokhirev, 2024) and they have not yet been thoroughly validated as clinical surrogate endpoints. Although technical noise can be minimized by utilizing approaches like principal component analysis (Higgins-Chen et al., 2022; Shokhirev and Johnson, 2025a), platform-specific noise can cause epigenetic age to fluctuate. Moreover, biological events like circadian rhythms (Koncevicius et al., 2024) or acute stress (Sehgal et al., 2025a) can cause temporary changes in epigenetic clocks. Cell-type composition is also relevant and a variable that influences age prediction (Guo and Teschendorff, 2025). As such, a change in an epigenetic aging clock in response to an intervention is not necessarily indicative of responsiveness or an improved clinical outcome. It could, for example, be a result of technical and/or biological noise.

In 2022, we published a review that listed interventional papers in humans involving aging clocks (Johnson et al., 2022). At that time, nine papers were identified and only three of these involved a next-generation epigenetic aging clock. Given that these newer clocks more strongly pick up on all-cause mortality risk, interventions that decelerate them are of interest. As such, we decided to update the list of human interventional research that includes at least one next-generation epigenetic aging clock. Here, we systematically collate 41 human interventional studies and detail which clocks were included, which ones were affected, and the effect size for any significant results. We additionally note how frequently distinct clocks were utilized and highlight performance differences in terms of responsiveness.

## Systematic search-based review to identify human interventional studies utilizing next-generation epigenetic clocks

### Next-generation clocks to search for

In a recent publication, first-versus next-generation aging clocks were defined and a comprehensive list of next-generation models was provided (Johnson and Shokhirev, 2025). In alphabetical order, these clocks are bAge (Bernabeu et al., 2023), CausAge (Ying et al., 2024), CheekAge (Shokhirev et al., 2024), DNAm PhenoAge (Levine et al., 2018), DunedinPACE (Belsky et al., 2022), DunedinPoAm (Belsky et al., 2020), FitAge (McGreevy et al., 2023), GrimAge (Lu et al., 2019), GrimAge2 (Lu et al., 2022), InflammAge (Schmunk et al., 2025), OMICmAge (Chen et al., 2026), and Systems Age (Sehgal et al., 2025b). All have been shown to be significantly associated with all-cause mortality risk in longitudinal data (Johnson and Shokhirev, 2025). As a note, the clocks AdaptAge and DamAge are spin-outs from CausAge and were designed to respectively capture beneficial, adaptive changes and damaging, harmful changes (Ying et al., 2024). There are also principal component (PC) versions of DNAm PhenoAge and GrimAge known as PC DNAm PhenoAge and PC GrimAge, respectively (Higgins-Chen et al., 2022).

### Criteria and exclusionary filters

The goal of our search was to identify interventional studies in humans that involved at least one next-generation epigenetic aging clock. In terms of criteria, we required that next-generation epigenetic age measurements must have been made pre- and post-intervention. Because of this, any study that only included first-generation models was excluded. In addition, observational and cross-sectional studies that did not explicitly involve an intervention were also omitted. Literature reviews, non-human animal studies, *in vitro* findings, as well as resource papers that cite previously published interventional studies were additionally excluded. Any paper published before 2018, which is when the first next-generation epigenetic aging clock was published (Levine et al., 2018), was also ignored. Preprints were considered acceptable so long as they met these criteria.

### Identifying published human interventions involving next-generation epigenetic aging clocks

To identify clinical trials involving next-generation clocks, we first searched for each model in quotes in PubMed and then filtered using the “Clinical Trial” article type. For example, searching for “GrimAge2” and applying the clinical trial filter only yielded a single result while searching for “DunedinPACE” with this filter yielded 10 results. Supplementary Table S1 lists all the PubMed results generated for each search. The following searches were applied in PubMed with this filter: “AdaptAge”, “bAge”, “CausAge”, “CheekAge”, “DunedinPACE”, “DunedinPoAm”, ‘FitAge”, “GrimAge”, “GrimAge2”, “InflammAge”, “OMICmAge”, “PhenoAge”, and “Systems Age”. Since “DamAge” returned an inordinate number of results and is consistently reported in combination with AdaptAge and/or CausAge, this was omitted. After removing duplicated PubMed IDs and manually reviewing each unique paper, a total of 15 papers were identified with this approach (Supplementary Table S1). Manual review entailed reading the title and abstract to determine whether or not the study represents an interventional investigation that meets our criteria.

**FIGURE 1:**
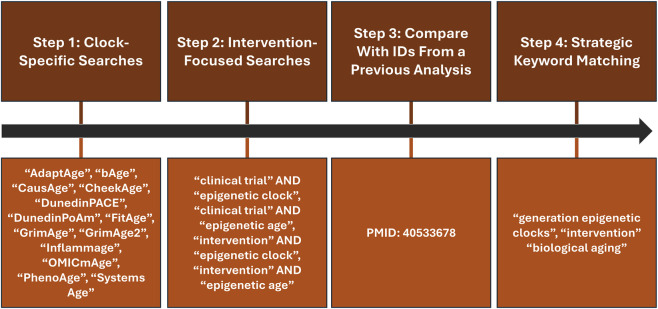
Systematic search approach employed to identify human interventional studies involving next-generation epigenetic aging clocks.

To identify additional interventional studies, the PubMed searches “clinical trial” AND “epigenetic clock”, “clinical trial” AND “epigenetic age”, “intervention” AND “epigenetic clock’, and “intervention” AND “epigenetic age” were performed. A much larger number of results were generated and, after combining them with unique IDs from the prior search, removing duplicates, and omitting non-interventional studies following manual review, a total of 36 PubMed IDs remained (Supplementary Table S1). After combing through studies identified in a previous literature analysis (Johnson and Shokhirev, 2025), searching for “generation epigenetic clocks”, and searching for “intervention” “biological aging” with a clinical trial filter, five additional papers were identified. The final result was 41 unique PubMed IDs involving next-generation epigenetic aging clocks (Supplementary Table S1). The search flow utilized to identify these studies is summarized visually in Figure 1.

### Collating results and assessing significance

While reviewing various studies and collating them in the primary tables (discussed below), it became clear that there were inconsistencies in terms of how groups reported significance. To address this, we only considered a result significant when its p-value (or q-value, if relevant) was below 0.05 and when the comparison was being made at the end of the intervention. For example, some studies included sampling along the way while the intervention was still being applied. If a significant result was reported after 3 months but not after 12 months for a 12-month intervention, this was not considered significant. Moreover, significance was only assessed for an entire interventional group, as opposed to isolated subgroups (e.g., individuals that were one standard deviation or higher at baseline for a given clock). The subject number was based on the number of subjects that completed the intervention as opposed to the number initially enrolled.

## Published interventional data in humans

### Significant pharmaceutical findings

**FIGURE 2:**
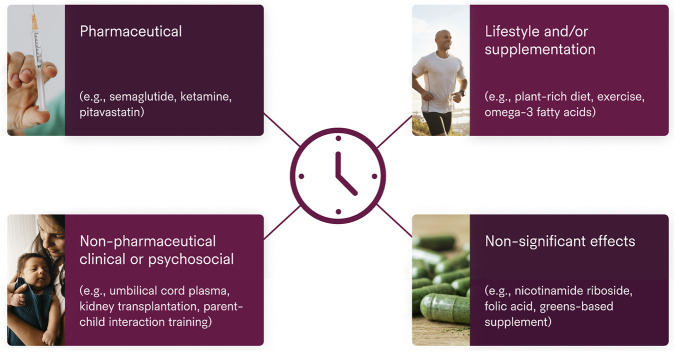
Overview of the types of interventions that have been reported to set back next-generation epigenetic aging clocks in humans.

We divided interventional studies into four different categories: pharmaceutical, lifestyle and/or supplementation, non-pharmaceutical clinical or psychosocial, and non-significant (Figure 2). Beginning with the first category, the following ten different pharmaceutical interventions have been reported to significantly change at least one next-generation epigenetic aging clock: emtricitabine-tenofovir-alafenamide, FTC-tenofovir-disoproxil fumarate, pitavastatin, decitabine, semaglutide, ketamine infusions, bezisterim, metformin, antiretroviral therapy, and a combination of growth hormone, dehydroepiandrosterone, and metformin (Table 1).

**TABLE 1:** Human studies reporting that a pharmaceutical intervention can significantly alter at least one next-generation epigenetic aging clock. Rows are sorted chronologically, from newest to oldest. Studies published in the same year are sorted alphabetically.

Intervention	Duration	Next-generation clocks tested	Subject number	Subject status	References
Emtricitabine-tenofovir-alafenamide	12 weeks	AdaptAge: ↓ DamAge: X DNAm PhenoAge: ↓ DunedinPACE: ↓ GrimAge: X GrimAge2: X OMICmAge: X PC DNAm PhenoAge: X PC GrimAge: X Systems Age: ↓	36	Healthy without HIV	Anderson et al. (2026)
FTC-tenofovir-disoproxil fumarate	12 weeks	AdaptAge: X DamAge: X DNAm PhenoAge: X DunedinPACE: X GrimAge: X GrimAge2: X OMICmAge: ↑ PC DNAm PhenoAge: X PC GrimAge: X Systems Age: X	43	Healthy without HIV	Anderson et al. (2026)
Pitavastatin	Two years	DunedinPACE: ↓ PC GrimAge: X	99	HIV	Corley et al. (2026)
Decitabine	Five days	AdaptAge: ↓ DamAge: ↑	28	Acute myeloid leukemia	Yan et al. (2026)
Semaglutide	32 weeks	AdaptAge: X CausAge: X DamAge: X DNAm PhenoAge: ↓ DunedinPACE: ↓ FitAge: X OMICmAge: ↓ PC DNAm PhenoAge: ↓ PC GrimAge: ↓ Systems Age: ↓	84	HIV-associated lipohypertrophy	Corley et al. (2025)
Ketamine infusions*	Two-three weeks	DNAm PhenoAge: X DunedinPACE: X GrimAge2: X OMICmAge: ↓ PC DNAm PhenoAge: X PC GrimAge: X Systems Age: X	20	Major depressive disorder or post-traumatic stress disorder	Dawson et al. (2025)
Bezisterim	30 weeks	DNAm PhenoAge: X GrimAge: X InflammAge: ↓	33	Mild-to-moderate probable Alzheimer’s disease	Reading et al. (2025)
Metformin	24 weeks	DunedinPACE: X PC DNAm PhenoAge: ↓ PC GrimAge: ↓	11	Virologically suppressed HIV with normal glucose	Corley et al. (2024)
Antiretroviral therapy	96 weeks	DNAm PhenoAge: ↓ GrimAge: ↓	168	HIV	Esteban-Cantos et al. (2021)
Growth hormone, dehydroepiandrosterone, and metformin	One year	DNAm PhenoAge: ↓ GrimAge: ↓	9	Healthy	Fahy et al. (2019)

* While the p-values for DNAm, PhenoAge, GrimAge2, and OMICmAGe were all below 0.05, only OMICmAge had a PCA, adjusted Bonferroni value under 0.05.

For example, one preprint study gave either the GLP-1 receptor agonist semaglutide or a placebo to 84 patients with HIV-associated lipohypertrophy for 32 weeks (Corley et al., 2025). For semaglutide, which was given once-weekly subcutaneously, patients started with an 8-week dose titration and then received a 1.0 mg dose for the following 24 weeks. Compared to the placebo group, semaglutide had striking effects on a variety of next-generation epigenetic aging clocks. Semaglutide treatment lowered annual epigenetic age relative to placebo by −4.9 years for DNAm PhenoAge, −2.2 years for OMICmAge, −3.68 years for PC DNAm PhenoAge, −3.08 years for PC GrimAge, and −4.17 years for Systems Age. DunedinPACE was also lowered by −0.09, reflecting a 9% drop in the annual pace of epigenetic aging. In contrast, no significant effects were reported for AdaptAge, CausAge, DamAge, and FitAge.

In a separate study, six ketamine infusions (0.5 mg/kg) were given to 20 subjects with post-traumatic stress disorder or major depressive disorder over the course of 2 to 3 weeks. Compared to baseline, OMICmAge dropped by −1.81 years. Although DNAm PhenoAge and GrimAge2 initially showed significant reductions, they were rendered insignificant after applying for a PCA adjusted Bonferroni correction. DunedinPACE, PC DNAm PhenoAge, PC GrimAge, and Systems Age were also not statistically significant.

The full list of pharmaceutical interventions and the reported effect sizes across all categories are in Table 1 and Supplementary Table S2, respectively. It is worth noting that decitabine–a cytidine analog used to treat myelodysplastic syndromes–dramatically decreased AdaptAge and increased DamAge (Yan et al., 2026). This suggests that the treatment may have negatively impacted adaptive epigenetic signals while increasing methylation-based damage signals. Similarly, a preprint study involving nucleos(t)ide reverse transcriptase inhibitors found that FTC-tenofovir-disoproxil fumarate increased OMICmAge and emtricitabine-tenofovir-alafenamide decreased AdaptAge when given to healthy individuals without HIV (Anderson et al., 2026).

### Significant lifestyle and/or supplementation findings

Ten different lifestyle and/or supplementation interventions (Table 2) have been reported to significantly alter next-generation models and include a multivitamin-multimineral supplement, supervised exercise and nutritional supplementation, cycling-based endurance exercise, supplementation in combination with encouragement to walk and practice mindfulness, a protein-energy supplement, caloric restriction, and a plant-rich diet. Three different studies have examined the impact of omega-3 fatty acids on epigenetic age, including one that gave omega-3 on its own, another that paired omega-3 with vitamin D, and a third that administered omega-3 by itself or in combination with vitamin D and an at-home exercise program.

**TABLE 2:** Human studies reporting that a supplement-based and/or lifestyle intervention can significantly alter at least one next-generation epigenetic aging clock. Rows are sorted chronologically, from newest to oldest. Studies published in the same year are sorted alphabetically.

Intervention	Duration	Next-generation clocks tested	Subject number	Subject status	References
Multivitamin-multimineral supplement and/or cocoa extract*	Two years	DunedinPACE: X PC DNAm PhenoAge: ↓ PC GrimAge: ↓	958	Healthy	Li et al. (2026)
Supervised exercise and nutritional supplementation	Six months	DNAm PhenoAge: ↓ FitAge: X GrimAge2: X	24	Frail	Olaso-Gonzalez et al. (2026)
Cycling-based endurance exercise	Six months	PC GrimAge: ↓	33	Healthy	Van Damme et al. (2026)
Vitamin D and/or omega-3 fatty acids and/or an at-home exercise program**	Over 3 years	DunedinPACE: ↓ FitAge: X GrimAge2: ↓ PC DNAm PhenoAge: ↓ PC GrimAge: X	777	Healthy	Bischoff-Ferrari et al. (2025)
Supplementation in combination with encouragement to walk and practice mindfulness***	One year	AdaptAge: X CausAge: X DamAge: X DunedinPACE: ↑ FitAge: X OMICmAge: X PC DNAm PhenoAge: X PC GrimAge: X	40	Healthy	Carreras-Gallo et al. (2025)
Protein-energy supplement	1,000 days (from conception until age two)	DNAm PhenoAge: ↓ DunedinPACE: ↓ GrimAge: ↓	1095	Young child (during the time of intervention)	Chapnick et al. (2025)
Vitamin D and/or omega-3 fatty acids****	Two years	DNAm PhenoAge: ↓ DunedinPACE: X GrimAge: ↓ GrimAge2: X	45	Mild cognitive impairment	Minami et al. (2025)
Omega-3 fatty acids	Six weeks	AdaptAge: ↑ CausAge: X DamAge: ↓ DNAm PhenoAge: X DunedinPACE: X GrimAge: X	69	Overweight	Ying et al. (2024)
Caloric restriction	Two years	DNAm PhenoAge: X DunedinPACE: ↓ GrimAge: X PC DNAm PhenoAge: X PC GrimAge: X	185	Non-obese	Waziry et al. (2023)
Plant-rich diet and/or increased physical activity*****	24 months	GrimAge: ↓	219	Healthy	Fiorito et al. (2021)

* In this study, only the multivitamin-multimineral supplement affected clocks. Cocoa extract did not significantly impact any of the measured clocks.

** While omega-3 significantly affected different clocks on its own, the three interventions together additively impacted PC DNAm PhenoAge.

*** Separate significant effects were reported for the next-generation clocks CausAge, DamAge, DunedinPACE, FitAge, OMICmAge, and PC GrimAge when making comparisons other than baseline versus 12 months, specifically baseline vs. three months, baseline versus 6 months, 3 months versus 12 months, or 6 months versus 12 months.

**** While GrimAge2 had a p-value less than 0.05, its q-value was greater than 0.05.

*****GrimAge was only reduced by the plant-rich diet intervention.

For the first omega-3 fatty acid study, Ying et al. reported that supplementation with omega-3 fatty acids for 6 weeks decreased DamAge by −6.1 years and increased AdaptAge by 6.2 years in a cohort of 69 overweight subjects (Ying et al., 2024). In 45 subjects with mild cognitive impairment, treatment with omega-3 fatty acids and/or vitamin D3 for 2 years was shown to drop DNAm PhenoAge by −1.61 years and GrimAge by −0.95 years (Minami et al., 2025). In a separate especially impressive study, 777 healthy people were given omega-3 fatty acids alone and/or vitamin D3 and/or an at-home exercise program for over 3 years. By itself, omega-3 fatty acids showed significant standardized estimates of −0.17 for DunedinPACE, −0.32 for GrimAge, and −0.16 for PC DNAm PhenoAge. These translated to a reduction of −0.0022 units/year for DunedinPACE, −0.64 years for GrimAge2, and -0.24 years for PC DNAm PhenoAge (Bischoff-Ferrari et al., 2025). A combination of omega-3 fatty acids, vitamin D, and exercise additively lowered PC DNAm PhenoAge by −0.32 years (Bischoff-Ferrari et al., 2025). These consistent results are intriguing given that omega-3 fatty acids represent essential nutrients that have been reported to attenuate inflammation and improve immune function (Poggioli et al., 2023).

More recently, Li et al. looked at methylomic data from 958 healthy older subjects that were given a multivitamin-multimineral supplement and/or cocoa extract for 2 years (Li et al., 2026). While cocoa extract had no effect, the daily multivitamin-multimineral supplement reduced PC DNAm PhenoAge by −0.44 years and PC GrimAge by −0.21 years relative to the placebo group. When expressed in terms of yearly change, the multivitamin-multimineral supplement caused a yearly drop of −0.21 years for PC DNAm PhenoAge and −0.11 years for PC GrimAge.

Significant effects have also been reported for lifestyle-specific interventions. In one recent paper, for example, 33 healthy individuals were given a cycling-based endurance exercise for 6 months. Compared to their baseline, PC GrimAge dropped by −0.62 years (Van Damme et al., 2026). In a separate study, 219 healthy individuals were assigned to a plant-rich diet and/or physical activity for 24 months. Relative to controls, the plant-rich diet arm displayed a −0.66 years reduction in GrimAge (Fiorito et al., 2021). Caloric restriction in 185 subjects also decreased DunedinPACE by 2%–3% at the 12-month and 24-month marks (Waziry et al., 2023). These results are very much aligned with broader evidence indicating that both physical activity and a high-quality, balanced diet can mitigate established aging hallmarks (Garatachea et al., 2015; Shannon et al., 2021).

Interestingly, one study found that supplementation in combination with encouragement to walk and practice mindfulness increased DunedinPACE by 0.050 after 1 year (Carreras-Gallo et al., 2025). Compared to baseline, 12 months of this intervention failed to impact AdaptAge, CausAge, DamAge, FitAge, OMICmAge, PC DNAm PhenoAge, and PC GrimAge. Additional significant results did, however, emerge when comparing other timepoints. For example, mean PC GrimAge went from 0.37 at the 6-month mark to −0.020 at the 12-month mark and this difference was statistically significant (Carreras-Gallo et al., 2025). All of these lifestyle and/or supplementation interventions, including a protein-energy supplement preprint study (Chapnick et al., 2025), are summarized in Table 2.

### Significant non-pharmaceutical clinical or psychosocial findings

While not as abundant, three significant non-pharmaceutical clinical therapeutics and one significant psychosocial intervention was found (Table 3). For the latter, an internet-based training program for parent-child interaction was given to 71 young developmentally delayed subjects for 20 weeks (Merrill et al., 2024). At the end of the intervention, DunedinPACE was reduced by −0.05 relative to controls. No other next-generation clocks were assessed in the study.

**TABLE 3:** Human studies reporting that a non-pharmaceutical clinical therapy or psychosocial intervention can significantly alter at least one next-generation epigenetic aging clock. Rows are sorted chronologically, from newest to oldest. Studies published in the same year are sorted alphabetically.

Intervention	Duration	Next-generation clocks tested	Subject number	Subject status	References
Multiple rounds of plasmaphereses	18 weeks	DNAm PhenoAge: X DunedinPACE: ↑ GrimAge: ↑ GrimAge2: ↑ PC DNAm PhenoAge: X PC GrimAge: X	34	Healthy	Borsky et al. (2025)
Internet-based training program for parent-child interaction	20 weeks	DunedinPACE: ↓	71	Developmental delay	Merrill et al. (2024)
Kidney transplantation or dialysis*	One year	DNAm PhenoAge: ↓	47	Chronic kidney disease	Neytchev et al. (2024)
Umbilical cord plasma concentrate	10 weeks	AdaptAge: X CausAge: X DamAge: ↓ DNAm PhenoAge: X DunedinPACE: X GrimAge: ↓	18	Normal health	Ying et al. (2024)
Umbilical cord plasma concentrate	10 weeks	DNAm PhenoAge: X GrimAge: ↓	18	Normal health	Clement et al. (2022)

* The post-intervention measurement was made 1 year after kidney transplantation or the initiation of dialysis. Kidney transplantation, but not dialysis, significantly lowered DNAm PhenoAge.

Regarding the non-pharmaceutical clinical therapeutics, these were multiple rounds of plasmaphereses, kidney transplantation, and umbilical cord plasma concentrate. Four to eight rounds of plasmaphereses over the course of 18 weeks in 34 healthy subjects actually led to significant increases in DunedinPACE (+0.003), GrimAge (+0.26), and GrimAge2 (+0.22) (Borsky et al., 2025). In contrast, kidney transplantation led to a −4.4 years decline in DNAm PhenoAge a year later in patients with chronic kidney disease while dialysis had no significant effect (Neytchev et al., 2024). Two different studies looked at the same dataset involving 18 older subjects of normal health that were given umbilical cord plasma concentrate for 10 weeks. The first study showed that there was no impact on DNAm PhenoAge but that GrimAge was lowered by −0.82 years compared to baseline (Clement et al., 2022). The latter study additionally looked at AdaptAge, CausAge, DamAge, and DunedinPACE. While AdaptAge, CausAge, and DunedinPACE were not significantly affected, DamAge did significantly drop by 2.4 years (Ying et al., 2024). Additional details for the studies in this category are in Table 3.

### Non-significant findings

The largest category was non-significant effects, which included 18 different studies and interventions (Table 4): S-adenosylmethionine and trauma-focused therapy, metformin, a greens-based supplement, rapamycin and home-based exercise, diet-based weight loss comparing low-fat and low-carbohydrate diets, antiretroviral therapy alone or a combination with candidate anti-HIV reservoir strategies, a relationship education program, dasatinib and quercetin or dasatinib, quercetin, and fisetin, a multi-component supplement, folic acid, nicotinamide riboside, a polyphenol concentrate supplement, sertraline and/or prolonged exposure therapy, an exercise program in conjunction with chemotherapy, various dietary approaches (healthy dietary guidelines, a Mediterranean diet, or a Green-Med diet), a substance-use prevention program, metformin and/or weight loss, and dietary counseling combined with resistance/aerobic exercise (Table 4). There are several reasons that a finding may be non-significant, such as the duration of the intervention being too short and limited statistical power that could not rise beyond technical and/or biological variability.

**TABLE 4:** Human interventional studies involving at least one next-generation epigenetic aging clock that reported non-significant results. Rows are sorted chronologically, from newest to oldest. Studies published in the same year are sorted alphabetically.

Intervention	Duration	Next-generation clock(s) tested	Subject #	Subject number	References
S-adenosylmethionine and trauma-focused therapy	12 weeks	DunedinPACE: X PC GrimAge: X PC DNAm PhenoAge: X	28	Trauma-related depression	Alkema et al. (2026)
Metformin	96 weeks	DNAm Phenoage: X DunedinPACE: X GrimAge2: X PC GrimAge: X PC DNAm PhenoAge: X	35	Non-diabetic with HIV	Marcelo-Calvo et al. (2026)
Greens-based supplement	30 days	AdaptAge: X DamAge: X PC GrimAge: X	19	BMI >30 kg/m^2^	Robinson et al. (2026)
Rapamycin and home-based exercise	13 weeks	DunedinPACE: X OMICmAge: X PC GrimAge: X Systems Age: X	33	Sedentary	Stanfield et al. (2026)
Diet-based weight loss comparing low-fat and low-carbohydrate diets	12 months	DunedinPACE: X PC DNAm PhenoAge: X PC GrimAge: X	112	Obese	Kou et al. (2025)
Antiretroviral therapy alone or a combination with candidate anti-HIV reservoir strategies	48 weeks	DNAm PhenoAge: X GrimAge: X	26	HIV with chronic disease	Ndhlovu et al. (2025)
Relationship education program	Six weeks	GrimAge: X	383	Individuals in a relationship	Weber et al. (2025)
Dasatinib and quercetin or dasatinib, quercetin, and fisetin	Six months	DunedinPACE: X PC DNAm PhenoAge: X PC GrimAge: X	19	Healthy	Lee et al. (2024)
Multi-component supplement*	12 weeks	InflammAge: X	80	Healthy	McGee et al. (2024)
Folic acid	Eight weeks	DNAm PhenoAge: X DunedinPACE: X GrimAge: X GrimAge2: X	16	Healthy	Michels and Binder (2024)
Nicotinamide riboside	10 weeks	DNAm PhenoAge: X GrimAge: X	20	Mild cognitive impairment	Orr et al. (2024)
Polyphenol concentrate supplement**	90 days	DunedinPACE: X OMICmAge: X PC DNAm PhenoAge: X PC GrimAge: X	40	Healthy	Perlmutter et al. (2024)
Sertraline and enhanced medication management, prolonged exposure therapy and placebo, or sertraline and prolonged exposure therapy	24 weeks	GrimAge: X	109	Post-traumatic stress disorder	Katrinli et al. (2023)
An exercise program over two rounds of chemotherapy	8–12 weeks	DNAm PhenoAge: X DunedinPACE: X GrimAge: X	20	Myeloid malignancies	Loh et al. (2023)
Healthy dietary guidelines, Mediterranean diet, or the Green-MED diet	18 months	DNAm PhenoAge: X DunedinPACE: X PC GrimAge: X	256	Abdominal obesity or dyslipidemia	Yaskolka Meir et al. (2023)
Substance-use prevention program	Six weeks	GrimAge: X	216	Rural youth	Lei et al. (2022)
Metformin and/or weight loss	Six months	DNAm PhenoAge: X GrimAge: X	192	Overweight/obese breast cancer survivors	Nwanaji-Enwerem et al. (2021)
Dietary counseling combined with resistance/aerobic exercise	12 weeks	DNAm PhenoAge: X	16	Obesity	Petersen et al. (2021)

* Although DNAm, PhenoAge and GrimAge were calculated, they were merged into a composite score alongside the Horvath 2013 and Hannum 2013 clocks. Moreover, InflammAge was significantly reduced when filtering for individuals with a delta age ≥ 2 years at baseline.

** Significant results were reported for OMICmAge, PC DNAm, PhenoAge, and PC GrimAge when subjects were stratified based on their initial delta age.

It is interesting to note that metformin failed to significantly alter epigenetic age in a study involving 192 overweight/obese breast cancer survivors and a treatment period of 6 months (Nwanaji-Enwerem et al., 2021). It also had no impact on various next-generation clocks when given to 35 non-diabetic people with HIV for 96 weeks (Marcelo-Calvo et al., 2026). In contrast, metformin given for 24 weeks significantly decreased PC GrimAge and PC DNAm PhenoAge in 11 people with virologically suppressed HIV and normal glucose (Corley et al., 2024). Another small study involving nine healthy men reported that both DNAm PhenoAge and GrimAge decreased after a 12-month intervention of growth hormone, dehydroepiandrosterone, and metformin (Fahy et al., 2019). Since these represent different populations and sample sizes (Tables 1, 4), additional research is needed to better understand the ability of metformin to influence validated epigenetic aging clocks.

### Interventional effect sizes

While all the effect sizes are noted in Supplementary Table S2, each significant effect is broken out by intervention and clock in Supplementary Table S3. In addition, the results are sorted based on the effect size. It is important to emphasize that the reported effect sizes are model-derived estimates that do not directly translate into a change in whole-body biological aging or expected lifespan. Disparate effect sizes are also likely to reflect differences in the underlying population being studied. Ultimately, one would expect that an aging biomarker would have more capacity to change in a less healthy cohort.

**FIGURE 3:**
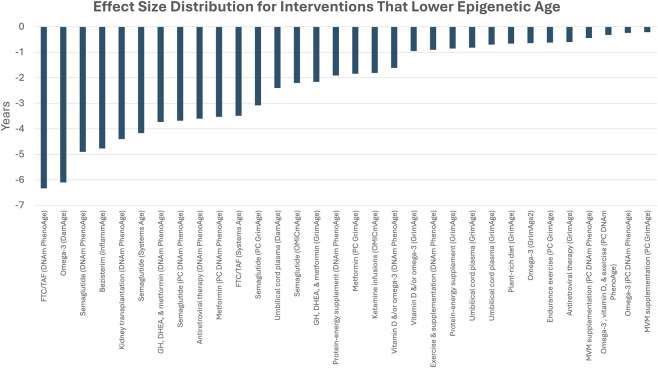
Visualization of the reported effect sizes for interventions that significantly set back next-generation clocks that output an age. FTC/TAF = Emtricitabine-tenofovir-alafenamide; GH = growth hormone; DHEA = dehydroepiandrosterone, MVM = multivitamin-multimineral supplement.

Among the interventions that positively influence next-generation clocks that output an age, the spread is quite sizeable (Figure 3). The largest impact was seen for emtricitabine-tenofovir-alafenamide, which lowered DNAm PhenoAge by 6.33 years. The next five largest favorable effect sizes were omega-3 fatty acids (−6.1 years and DamAge), semaglutide (−4.9 years and DNAm PhenoAge), bezisterim (−4.77 years and InflammAge), kidney transplantation (−4.4 years and DNAm PhenoAge), and semaglutide (−4.17 years and Systems Age). For the rate of aging clock DunedinPACE, the most prominent beneficial impact was seen for semaglutide, which lowered DunedinPACE by 9%. The next largest favorable effect sizes were −0.061 (emtricitabine-tenofovir-alafenamide), −0.05 (internet-based training program for parent-child interaction), −0.035 (pitavastatin), −0.03 (protein-energy supplement), −0.025 (caloric restriction), and −0.0022 (omega-3 fatty acids).

For interventions that alter clocks in a direction that would be expected to be deleterious, both AdaptAge and DamAge were highly impacted by decitabine in patients with acute myeloid leukemia. Plasmapheresis modestly elevated DunedinPACE (+0.003), GrimAge (+0.26), and GrimAge2 (+0.22), emtricitabine-tenofovir-alafenamide decreased AdaptAge by −5.92 years, and FTC-tenofovir-disoproxil fumarate increased OMICmAge by 0.63 years. Supplementation in combination with encouragement to walk and practice mindfulness led to a 0.05 increase in DunedinPACE (Supplementary Table S3).

### Next-generation clock usage and comparison

Finally, we examined the frequency at which different next-generation clocks were used in human interventional studies (Supplementary Table S4). Not surprisingly, two of the most used models were the earliest next-generation epigenetic aging clocks, DNAm PhenoAge and GrimAge. DNAm PhenoAge and GrimAge were assessed in 24 and 21 interventions, respectively. The most included clock was DunedinPACE, which was measured in 25 different interventions (Supplementary Table S4).

In addition to how many times each clock appeared in the primary tables (Tables 1-4), the number of times the clock was significantly affected and the percentage of times it responded when measured is also noted in Supplementary Table S4. Looking at clocks that were included 10 or more times, the most responsive models were GrimAge (38.1% responsiveness), DunedinPACE (36% responsiveness), and DNAm PhenoAge (33.3% responsiveness). Although FitAge and CausAge were included in four different interventions, they did not significantly respond to any of the treatments (Supplementary Table S4).

## Concluding remarks

An ambitious goal of the biohorology field is to develop aging biomarkers and clocks that can predict human lifespan accurately enough to act as surrogates for human clinical trials (Ferrucci et al., 2020; Galkin et al., 2020; Cummings and Kritchevsky, 2022). Given how long humans live, lifespan studies in our species are impractical from both a time and cost perspective (Johnson, 2025). If aging could be quantified with sufficient precision, as many longevity scientists believe it can be (Cohen et al., 2020), the effects of interventions on human lifespan and overall health could be rapidly and efficiently tested.

In this systematic search-based review, we show that next-generation aging clocks are apparently slowed by well-established interventions, such as a high-quality, plant-rich diet and endurance exercise. We also demonstrate that commonly used drugs like a statin or a GLP-1 receptor agonist can decrease the age outputted by next-generation clocks. Other interventions, including omega-3 fatty acid supplementation, multivitamin-multimineral supplementation, caloric restriction, umbilical cord plasma, and ketamine infusions have also been reported to slow epigenetic aging clocks. A deeper dissection of what methylation changes mechanistically underlie these interventional responses is warranted.

To help drive the biohorology field forward, additional research is needed to strengthen the association of epigenetic clocks with age-related outcomes and enhance their ability to respond to beneficial interventions. It also remains to be determined if test-retest reproducibility can be substantially improved beyond what is currently capable with PC-based clocks. This is an important consideration as the more resistant a clock is to technical noise, the smaller the sample size that will be needed to detect a meaningful change.

Finally, interpretability may be especially valuable in a clinical context. If a clock is slowed, understanding what changed and why could help scientists and clinicians better understand the effects of an intervention. Recent advances in artificial intelligence have led to the development of more interpretable epigenetic clocks (Prosz et al., 2024; Kalyakulina et al., 2025; Shokhirev and Johnson, 2025b) and additional progress is expected.

## References

[B1] Alkema A. Schalkwijk W. Bohte E. Draisma L. Hoppe A. Koch C. (2026). S-adenosylmethionine as an epigenetic treatment of depression in adults with childhood trauma. Epigenomics 18, 1–13. 10.1080/17501911.2026.2645001 42027147 PMC13166236

[B2] Anderson P. L. Pang A. P. Coyle R. P. Schlachetzki J. Molina A. J. A. Bushman L. (2026). An FDA-approved Tenofovir Alafenamide-Based antiretroviral therapy reduces biological Age in healthy adults: first human proof-of-concept for Retrotransposon-Targeted gerotherapeutics. 10.64898/2026.03.23.26349105

[B3] Belsky D. W. Caspi A. Arseneault L. Baccarelli A. Corcoran D. L. Gao X. (2020). Quantification of the pace of biological aging in humans through a blood test, the DunedinPoAm DNA methylation algorithm. Elife 9, e54870. 10.7554/eLife.54870 32367804 PMC7282814

[B4] Belsky D. W. Caspi A. Corcoran D. L. Sugden K. Poulton R. Arseneault L. (2022). DunedinPACE, a DNA methylation biomarker of the pace of aging. Elife 11, e73420. 10.7554/eLife.73420 35029144 PMC8853656

[B5] Bernabeu E. McCartney D. L. Gadd D. A. Hillary R. F. Lu A. T. Murphy L. (2023). Refining epigenetic prediction of chronological and biological age. Genome Med. 15 (1), 12. 10.1186/s13073-023-01161-y 36855161 PMC9976489

[B6] Bischoff-Ferrari H. A. Gangler S. Wieczorek M. Belsky D. W. Ryan J. Kressig R. W. (2025). Individual and additive effects of vitamin D, omega-3 and exercise on DNA methylation clocks of biological aging in older adults from the DO-HEALTH trial. Nat. Aging 5 (3), 376–385. 10.1038/s43587-024-00793-y 39900648 PMC11922767

[B7] Bocklandt S. Lin W. Sehl M. E. Sanchez F. J. Sinsheimer J. S. Horvath S. (2011). Epigenetic predictor of age. PLoS One 6 (6), e14821. 10.1371/journal.pone.0014821 21731603 PMC3120753

[B8] Borsky P. Holmannova D. Parova H. Horvath S. Sramek P. Brooke R. T. (2025). Human clinical trial of plasmapheresis effects on biomarkers of aging (efficacy and safety trial). Sci. Rep. 15 (1), 21059. 10.1038/s41598-025-05396-0 40592961 PMC12218284

[B9] Carreras-Gallo N. Dargham R. Thorpe S. P. Warren S. Mendez T. L. Smith R. (2025). Effects of a natural ingredients-based intervention targeting the hallmarks of aging on epigenetic clocks, physical function, and body composition: a single-arm clinical trial. Aging (Albany NY) 17 (3), 699–725. 10.18632/aging.206221 40096467 PMC11984428

[B10] Chapnick M. Yu E. A. Smith A. K. Conneely K. N. Ramirez-Zea M. Qin Z. (2025). Early-life nutrition and epigenetic age in middle-adulthood among Guatemalan adults. bioRxiv. 10.1101/2025.09.25.678652

[B11] Chen Q. Dwaraka V. B. Carreras-Gallo N. Armstrong J. F. Sehgal R. Argentieri M. A. (2026). OMICmAge quantifies biological age by integrating multi-omics with electronic medical records. Nat. Aging 6, 722–737. 10.1038/s43587-026-01073-7 41741793 PMC13004675

[B12] Clement J. Yan Q. Agrawal M. Coronado R. E. Sturges J. A. Horvath M. (2022). Umbilical cord plasma concentrate has beneficial effects on DNA methylation GrimAge and human clinical biomarkers. Aging Cell 21 (10), e13696. 10.1111/acel.13696 36052758 PMC9577957

[B13] Cohen A. A. Kennedy B. K. Anglas U. Bronikowski A. M. Deelen J. Dufour F. (2020). Lack of consensus on an aging biology paradigm? A global survey reveals an agreement to disagree, and the need for an interdisciplinary framework. Mech. Ageing Dev. 191, 111316. 10.1016/j.mad.2020.111316 32693105 PMC7603428

[B14] Corley M. J. Pang A. P. S. Shikuma C. M. Ndhlovu L. C. (2024). Cell-type specific impact of metformin on monocyte epigenetic age reversal in virally suppressed older people living with HIV. Aging Cell 23 (1), e13926. 10.1111/acel.13926 37675817 PMC10776116

[B15] Corley M. J. Dwaraka V. Pang A. P. Labbato D. Smith R. Eckard A. R. (2025). Semaglutide slows epigenetic aging in people with HIV-Associated lipohypertrophy: evidence from a randomized controlled trial. 10.1101/2025.07.09.25331038 PMC13381876 42156721

[B16] Corley M. J. Watanabe M. Pang A. P. S. Dwaraka V. B. Smith R. Samaneka W. (2026). Effect of pitavastatin on epigenetic aging biomarkers in people with HIV: pilot substudy of the REPRIEVE trial. Clin. Infect. Dis. 81 (6), e560–e567. 10.1093/cid/ciaf247 40576558 PMC12481469

[B17] Cummings S. R. Kritchevsky S. B. (2022). Endpoints for geroscience clinical trials: health outcomes, biomarkers, and biologic age. Geroscience 44 (6), 2925–2931. 10.1007/s11357-022-00671-8 36260264 PMC9768060

[B18] Dawson K. L. Carangan A. Klunder J. Carreras-Gallo N. Sehgal R. Megilligan S. (2025). Epigenetic aging and DNA methylation biomarker changes following ketamine treatment in patients with MDD and PTSD: a pilot study. Transl. Psychiatry 15 (1), 452. 10.1038/s41398-025-03683-y 41173838 PMC12579232

[B19] Esteban-Cantos A. Rodriguez-Centeno J. Barruz P. Alejos B. Saiz-Medrano G. Nevado J. (2021). Epigenetic age acceleration changes 2 years after antiretroviral therapy initiation in adults with HIV: a substudy of the NEAT001/ANRS143 randomised trial. Lancet HIV 8 (4), e197–e205. 10.1016/S2352-3018(21)00006-0 33794182

[B20] Fahy G. M. Brooke R. T. Watson J. P. Good Z. Vasanawala S. S. Maecker H. (2019). Reversal of epigenetic aging and immunosenescent trends in humans. Aging Cell 18 (6), e13028. 10.1111/acel.13028 31496122 PMC6826138

[B21] Ferrucci L. Gonzalez-Freire M. Fabbri E. Simonsick E. Tanaka T. Moore Z. (2020). Measuring biological aging in humans: a quest. Aging Cell 19 (2), e13080. 10.1111/acel.13080 31833194 PMC6996955

[B22] Fiorito G. Caini S. Palli D. Bendinelli B. Saieva C. Ermini I. (2021). DNA methylation-based biomarkers of aging were slowed down in a two-year diet and physical activity intervention trial: the DAMA study. Aging Cell 20 (10), e13439. 10.1111/acel.13439 34535961 PMC8520727

[B23] Galkin F. Mamoshina P. Aliper A. de Magalhaes J. P. Gladyshev V. N. Zhavoronkov A. (2020). Biohorology and biomarkers of aging: current state-of-the-art, challenges and opportunities. Ageing Res. Rev. 60, 101050. 10.1016/j.arr.2020.101050 32272169

[B24] Garatachea N. Pareja-Galeano H. Sanchis-Gomar F. Santos-Lozano A. Fiuza-Luces C. Moran M. (2015). Exercise attenuates the major hallmarks of aging. Rejuvenation Res. 18 (1), 57–89. 10.1089/rej.2014.1623 25431878 PMC4340807

[B25] Guo X. Teschendorff A. E. (2025). Epigenetic clocks and inflammaging: pitfalls caused by ignoring cell-type heterogeneity. Geroscience 47 (3), 2707–2719. 10.1007/s11357-025-01677-8 40299262 PMC12181471

[B26] Hannum G. Guinney J. Zhao L. Zhang L. Hughes G. Sadda S. (2013). Genome-wide methylation profiles reveal quantitative views of human aging rates. Mol. Cell 49 (2), 359–367. 10.1016/j.molcel.2012.10.016 23177740 PMC3780611

[B27] Higgins-Chen A. T. Thrush K. L. Wang Y. Minteer C. J. Kuo P. L. Wang M. (2022). A computational solution for bolstering reliability of epigenetic clocks: implications for clinical trials and longitudinal tracking. Nat. Aging 2 (7), 644–661. 10.1038/s43587-022-00248-2 36277076 PMC9586209

[B28] Horvath S. (2013). DNA methylation age of human tissues and cell types. Genome Biol. 14 (10), R115. 10.1186/gb-2013-14-10-r115 24138928 PMC4015143

[B29] Johnson A. A. (2025). Realistic expectations for changes to average human lifespan in the near future. Biogerontology 26 (5), 176. 10.1007/s10522-025-10318-8 40879822

[B30] Johnson A. A. Shokhirev M. N. (2024). Contextualizing aging clocks and properly describing biological age. Aging Cell 23 (12), e14377. 10.1111/acel.14377 39392224 PMC11634725

[B31] Johnson A. A. Shokhirev M. N. (2025). First-generation *versus* next-generation epigenetic aging clocks: differences in performance and utility. Biogerontology 26 (4), 121. 10.1007/s10522-025-10265-4 40533678

[B32] Johnson A. A. English B. W. Shokhirev M. N. Sinclair D. A. Cuellar T. L. (2022). Human age reversal: fact or fiction? Aging Cell 21 (8), e13664. 10.1111/acel.13664 35778957 PMC9381899

[B33] Kalyakulina A. Yusipov I. Trukhanov A. Franceschi C. Moskalev A. Ivanchenko M. (2025). EpInflammAge: Epigenetic-Inflammatory clock for disease-associated biological aging based on deep learning. Int. J. Mol. Sci. 26 (13), 6284. 10.3390/ijms26136284 40650062 PMC12249966

[B34] Katrinli S. King A. P. Duval E. R. Smith A. K. Rajaram N. Liberzon I. (2023). DNA methylation GrimAge acceleration in US military veterans with PTSD. Neuropsychopharmacology 48 (5), 773–780. 10.1038/s41386-023-01537-z 36725867 PMC10066228

[B35] Koncevicius K. Nair A. Sveikauskaite A. Sestokaite A. Kazlauskaite A. Dulskas A. (2024). Epigenetic age oscillates during the day. Aging Cell 23 (7), e14170. 10.1111/acel.14170 38638005 PMC11258449

[B36] Kou M. Li X. Heianza Y. Dorans K. Bazzano L. Qi L. (2025). Epigenetic Age acceleration and cardiometabolic biomarkers in response to weight-loss dietary interventions among Obese individuals: the MACRO trial. Aging Cell 24 (11), e70224. 10.1111/acel.70224 40922554 PMC12611269

[B37] Lee E. Carreras-Gallo N. Lopez L. Turner L. Lin A. Mendez T. L. (2024). Exploring the effects of Dasatinib, Quercetin, and Fisetin on DNA methylation clocks: a longitudinal study on senolytic interventions. Aging (Albany NY) 16 (4), 3088–3106. 10.18632/aging.205581 38393697 PMC10929829

[B38] Lei M. K. Brody G. H. Beach S. R. H. (2022). Intervention effects on self-control decrease speed of biological aging mediated by changes in substance use: a longitudinal study of African American youth. Fam. Process 61 (2), 659–673. 10.1111/famp.12715 34389984 PMC8841568

[B39] Levine M. E. Lu A. T. Quach A. Chen B. H. Assimes T. L. Bandinelli S. (2018). An epigenetic biomarker of aging for lifespan and healthspan. Aging (Albany NY) 10 (4), 573–591. 10.18632/aging.101414 29676998 PMC5940111

[B40] LiS. HamayaR. ZhuH. ChenB. H. PereiraA. C. IveyK. L. (2026). Effects of daily multivitamin-multimineral and cocoa extract on epigenetic aging clocks in the COSMOS randomized clinical trial. Nat. Med. 32, 1012–1022. 10.1038/s41591-026-04239-3 41803341

[B41] Loh K. P. Sanapala C. Jensen-Battaglia M. Rana A. Sohn M. B. Watson E. (2023). Exercise and epigenetic ages in older adults with myeloid malignancies. Eur. J. Med. Res. 28 (1), 180. 10.1186/s40001-023-01145-z 37254221 PMC10227405

[B42] Lu A. T. Quach A. Wilson J. G. Reiner A. P. Aviv A. Raj K. (2019). DNA methylation GrimAge strongly predicts lifespan and healthspan. Aging (Albany NY) 11 (2), 303–327. 10.18632/aging.101684 30669119 PMC6366976

[B43] Lu A. T. Binder A. M. Zhang J. Yan Q. Reiner A. P. Cox S. R. (2022). DNA methylation GrimAge version 2. Aging (Albany NY) 14 (23), 9484–9549. 10.18632/aging.204434 36516495 PMC9792204

[B44] Marcelo-Calvo C. Esteban-Cantos A. Jurado F. Montejano R. Rodriguez-Centeno J. Gutierrez-Garcia L. (2026). Metformin and epigenetic age in non-diabetic older people with HIV in Madrid (METFORAGING): a double-blind, randomised, placebo-controlled, pilot trial. EClinicalMedicine 95, 103874. 10.1016/j.eclinm.2026.103874 42023167 PMC13098334

[B45] McGee K. C. Sullivan J. Hazeldine J. Schmunk L. J. Martin-Herranz D. E. Jackson T. (2024). A combination nutritional supplement reduces DNA methylation age only in older adults with a raised epigenetic age. Geroscience 46 (5), 4333–4347. 10.1007/s11357-024-01138-8 38528176 PMC11336001

[B46] McGreevy K. M. Radak Z. Torma F. Jokai M. Lu A. T. Belsky D. W. (2023). DNAmFitAge: biological age indicator incorporating physical fitness. Aging (Albany NY) 15 (10), 3904–3938. 10.18632/aging.204538 36812475 PMC10258016

[B47] Merrill S. M. Hogan C. Bozack A. K. Cardenas A. Comer J. S. Bagner D. M. (2024). Telehealth Parenting Program and salivary epigenetic biomarkers in preschool children with developmental delay: NIMHD social epigenomics Program. JAMA Netw. Open 7 (7), e2424815. 10.1001/jamanetworkopen.2024.24815 39073812 PMC11287424

[B48] Michels K. B. Binder A. M. (2024). Impact of folic acid on the epigenetic profile in healthy unfortified individuals - a randomized intervention trial. Epigenetics 19 (1), 2293410. 10.1080/15592294.2023.2293410 38096372 PMC10730197

[B49] Minami K. Shirai T. Okazaki S. Okada S. Miyachi M. Otsuka I. (2025). Preliminary longitudinal epigenetic clock analyses of patients with mild cognitive impairment through nutritional intervention. J. Alzheimers Dis. 107 (1), 267–276. 10.1177/13872877251360230 40686203

[B50] Ndhlovu L. C. Giron L. B. Galinskas J. Premeaux T. A. Pang A. P. S. Dias D. (2025). Virological and immunological outcomes of combined therapeutic interventions and dendritic cell therapy in people with HIV. J. Infect. Dis. 232 (5), 1067–1077. 10.1093/infdis/jiaf430 40810569 PMC12614972

[B51] Neytchev O. Erlandsson H. Witasp A. Nordfors L. Qureshi A. R. Iseri K. (2024). Epigenetic clocks indicate that kidney transplantation and not dialysis mitigate the effects of renal ageing. J. Intern Med. 295 (1), 79–90. 10.1111/joim.13724 37827529

[B52] Nwanaji-Enwerem J. C. Chung F. F. Van der Laan L. Novoloaca A. Cuenin C. Johansson H. (2021). An epigenetic aging analysis of randomized metformin and weight loss interventions in overweight postmenopausal breast cancer survivors. Clin. Epigenetics 13 (1), 224. 10.1186/s13148-021-01218-y 34920739 PMC8684118

[B53] Olaso-Gonzalez G. Millan-Domingo F. Garcia-Fernandez L. Garcia-Tercero E. Cebrian M. Garcia-Dominguez C. (2026). A multidomain lifestyle intervention is associated with improved functional trajectories and favorable changes in epigenetic aging markers in frail older adults: a randomized controlled trial. Aging Cell 25 (2), e70376. 10.1111/acel.70376 41677077 PMC12895478

[B54] Orr M. E. Kotkowski E. Ramirez P. Bair-Kelps D. Liu Q. Brenner C. (2024). A randomized placebo-controlled trial of nicotinamide riboside in older adults with mild cognitive impairment. Geroscience 46 (1), 665–682. 10.1007/s11357-023-00999-9 37994989 PMC10828186

[B55] Perlmutter A. Bland J. S. Chandra A. Malani S. S. Smith R. Mendez T. L. (2024). The impact of a polyphenol-rich supplement on epigenetic and cellular markers of immune age: a pilot clinical study. Front. Nutr. 11, 1474597. 10.3389/fnut.2024.1474597 39628466 PMC11612904

[B56] Petersen C. L. Christensen B. C. Batsis J. A. (2021). Weight management intervention identifies association of decreased DNA methylation age with improved functional age measures in older adults with obesity. Clin. Epigenetics 13 (1), 46. 10.1186/s13148-021-01031-7 33653394 PMC7927264

[B58] Poggioli R. Hirani K. Jogani V. G. Ricordi C. (2023). Modulation of inflammation and immunity by omega-3 fatty acids: a possible role for prevention and to halt disease progression in autoimmune, viral, and age-related disorders. Eur. Rev. Med. Pharmacol. Sci. 27 (15), 7380–7400. 10.26355/eurrev_202308_33310 37606147

[B59] Prosz A. Pipek O. Borcsok J. Palla G. Szallasi Z. Spisak S. (2024). Biologically informed deep learning for explainable epigenetic clocks. Sci. Rep. 14 (1), 1306. 10.1038/s41598-023-50495-5 38225268 PMC10789766

[B60] Reading C. L. Yan J. Testa M. A. Simonson D. C. Javaid H. Schmunk L. (2025). An exploratory analysis of bezisterim treatment associated with decreased biological age acceleration, and improved clinical measure and biomarker changes in mild-to-moderate probable Alzheimer's disease. Front. Neurosci. 19, 1516746. 10.3389/fnins.2025.1516746 40386807 PMC12082838

[B61] Robinson L. A. Cavanah A. M. Lennon S. Mattingly M. L. Pol W. V. Huggins K. W. (2026). Epigenetic and microbiome responses to greens in obese older adults: results from a randomized crossover-controlled trial. Front. Nutr. 13, 1750030. 10.3389/fnut.2026.1750030 41717034 PMC12915338

[B62] Schmunk L. J. Call T. P. McCartney D. L. Javaid H. Hastings W. J. Jovicevic V. (2025). A novel framework to build saliva-based DNA methylation biomarkers: quantifying systemic chronic inflammation as a case study. Aging Cell 24 (4), e14444. 10.1111/acel.14444 39888134 PMC11984670

[B63] Sehgal R. Borrus D. Gonzalez J. Markov Y. Higgins-Chen A. (2025a). Biological *versus* technical reliability of epigenetic clocks and implications for disease prognosis and intervention response. bioRxiv [Preprint]. 2025.10.13.682176. 10.1101/2025.10.13.682176 PMC13418614 42525215

[B64] Sehgal R. Markov Y. Qin C. Meer M. Hadley C. Shadyab A. H. (2025b). Systems Age: a single blood methylation test to quantify aging heterogeneity across 11 physiological systems. Nat. Aging 5 (9), 1880–1896. 10.1038/s43587-025-00958-3 40954326 PMC13222069

[B65] Shannon O. M. Ashor A. W. Scialo F. Saretzki G. Martin-Ruiz C. Lara J. (2021). Mediterranean diet and the hallmarks of ageing. Eur. J. Clin. Nutr. 75 (8), 1176–1192. 10.1038/s41430-020-00841-x 33514872

[B66] Shokhirev M. N. Johnson A. A. (2025a). Analysis of variability and epigenetic age prediction across microarray and methylation sequencing technologies. Geroscience 47 (5), 6631–6638. 10.1007/s11357-025-01824-1 40784975 PMC12741009

[B67] Shokhirev M. N. Johnson A. A. (2025b). Using buccal methylomic data to create explainable aging clocks as well as classifiers and regressors for lifestyle and demographic factors. Front. Genet. 16, 1637186. 10.3389/fgene.2025.1637186 41104118 PMC12521809

[B68] Shokhirev M. N. Torosin N. S. Kramer D. J. Johnson A. A. Cuellar T. L. (2024). CheekAge: a next-generation buccal epigenetic aging clock associated with lifestyle and health. Geroscience 46 (3), 3429–3443. 10.1007/s11357-024-01094-3 38441802 PMC11009193

[B69] Stanfield B. Leroux B. Kaeberlein M. Jones J. Lucas R. (2026). Exercise and weekly sirolimus (Rapamycin) in older adults: RAPA-EX-01 randomised, Double-Blind, placebo-controlled trial. J. Cachexia Sarcopenia Muscle 17 (2), e70274. 10.1002/jcsm.70274 41985884 PMC13082878

[B70] Van Damme M. Stegen S. Steenwinckel B. Schroe H. Reyes Del Paso G. A. Pulopulos M. M. (2026). Epigenetic age deceleration reflects exercise-induced cardiorespiratory fitness improvements. Geroscience. 10.1007/s11357-025-02076-9 PMC13638861 41547677

[B71] Waziry R. Ryan C. P. Corcoran D. L. Huffman K. M. Kobor M. S. Kothari M. (2023). Effect of long-term caloric restriction on DNA methylation measures of biological aging in healthy adults from the CALERIE trial. Nat. Aging 3 (3), 248–257. 10.1038/s43587-022-00357-y 37118425 PMC10148951

[B72] Weber D. M. Lavner J. A. Carter S. Ong M. L. Lei M. K. Philibert R. (2025). Relationship intervention moderates the association between substance use and biological aging among Black adults. Health Psychol. 44 (9), 844–853. 10.1037/hea0001495 40167560 PMC12353662

[B73] Yan Z. Yuan L. Wang J. Gu S. Lei Y. (2026). PRC2-Related epigenetic Age acceleration in Acute Myeloid leukemia with DNMT3A and IDH2 mutations. Adv. Biol. (Weinh) 10 (1), e00710. 10.1002/adbi.202500710 41556261 PMC12817234

[B74] Yaskolka Meir A. Keller M. Hoffmann A. Rinott E. Tsaban G. Kaplan A. (2023). The effect of polyphenols on DNA methylation-assessed biological age attenuation: the DIRECT PLUS randomized controlled trial. BMC Med. 21 (1), 364. 10.1186/s12916-023-03067-3 37743489 PMC10519069

[B75] Ying K. Liu H. Tarkhov A. E. Sadler M. C. Lu A. T. Moqri M. (2024). Causality-enriched epigenetic age uncouples damage and adaptation. Nat. Aging 4 (2), 231–246. 10.1038/s43587-023-00557-0 38243142 PMC11070280

